# Early experience with single-incision laparoscopic surgery for the placement of a gastrostomy in a 10-year-old girl: a case report

**DOI:** 10.1186/1752-1947-6-375

**Published:** 2012-11-06

**Authors:** Kim Vanderlinden, Nele Van De Winkel, Antoine De Backer, Georges Delvaux, Kristel De Vogelaere

**Affiliations:** 1 Department of Abdominal and Pediatric Surgery, UZ Brussel, Brussels, Belgium

**Keywords:** Gastrostomy, Single-incision laparoscopic surgery, Single-port, Transumbilical

## Abstract

**Introduction:**

Access procedures for alimentation have been performed both endoscopically and surgically. In patients in whom endoscopic gastrostomy feeding tubes cannot be placed, single-incision laparoscopic surgery gastrostomy is an alternative method. This minimally invasive approach is a new technique performed through a single umbilical incision and without the need for additional laparoscopic ports.

**Case presentation:**

In this article we present a case of single-incision laparoscopic surgery gastrostomy performed with conventional laparoscopic instruments in a 10-year-old girl of Caucasian ethnicity who was not a candidate for a percutaneous endoscopic gastrostomy tube because of esophageal varices due to her advanced-stage cystic fibrosis with liver cirrhosis and portal hypertension. She also had an umbilical hernia, which was repaired during the same procedure through the same incision. Access and pneumoperitoneum were obtained through the umbilicus with the single-incision laparoscopic surgery port. The selected site for the feeding tube in the stomach was exteriorized through this incision and a feeding tube was placed. The stomach was returned into the abdomen. The fascial defect, and thus also the hernia, was repaired, and the 2cm umbilical incision was closed with endocutaneous sutures. The total operative time was 25 minutes. Our patient’s intra-operative and post-operative course was uneventful. We were able to use the feeding tube on the first post-operative day with good intestinal function. Our patient and her parents were pleased with the cosmetic result.

**Conclusions:**

The single-incision laparoscopic surgery procedure seems to be a less invasive alternative to open placement of gastrostomy. This approach has the possible advantages of reduced post-operative pain, faster return to normal function, reduced port site complications, improved cosmesis and better patient satisfaction.

## Introduction

As technology and innovation continue to advance in the field of minimally invasive surgery, the use of single-incision laparoscopic surgery (SILS) is gaining popularity. Single-port surgery is a procedure in which laparoscopic surgery is performed through a small incision at the umbilicus or another region in the abdomen. Applications have been previously described using this approach for various procedures in adults. In children, SILS procedures have been published in particular for cholecystectomy and appendectomy.

Potential advantages over conventional laparoscopic surgery include better cosmesis, less post-operative wound pain, and fewer port site complications.

## Case presentation

Our patient was a 10-year-old girl of Caucasian ethnicity. She had an advanced stage of cystic fibrosis with liver cirrhosis, portal hypertension and hepatosplenomegaly. She needed supplementary tube feeding because of weight loss due to external compression from her enlarged liver and spleen on the stomach. It was not possible to place a gastrostomy by gastroscopy because of esophageal varices. For this reason, a surgical approach was mandatory. Because of the enormous hepatosplenomegaly it would have been difficult to find the stomach under the left liver lobe by median laparotomy. For that reason we decided to use the SILS procedure. She also had an umbilical hernia with incarcerated fat, which was repaired during the same procedure.

**Figure 1:**
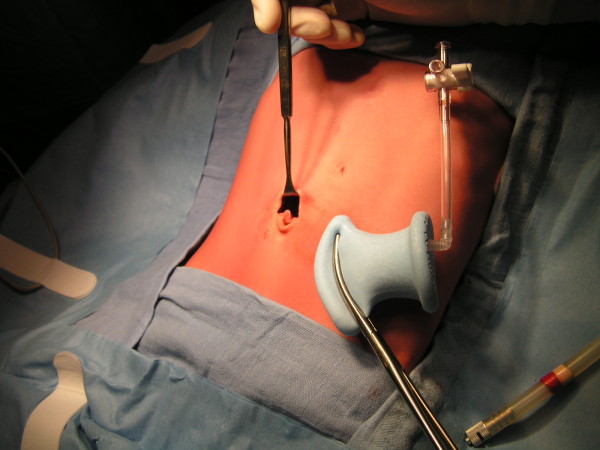
A 2cm hemi-circumferential incision was made at the upper half of the umbilical ring to insert the single-incision laparoscopic surgery port.

The operation was carried out under general anesthesia via endotracheal intubation with our patient in dorsal decubitus, tilted to a 30° anti-Trendelenburg position. After appropriate skin preparation and application of sterile drapes, a 2cm incision was made at the upper half of the umbilical ring, at the place of the hernia (Figure 1). The incision was deepened to the fascia. The hernial sac was dissected and opened so we could enter the abdomen.

**Figure 2:**
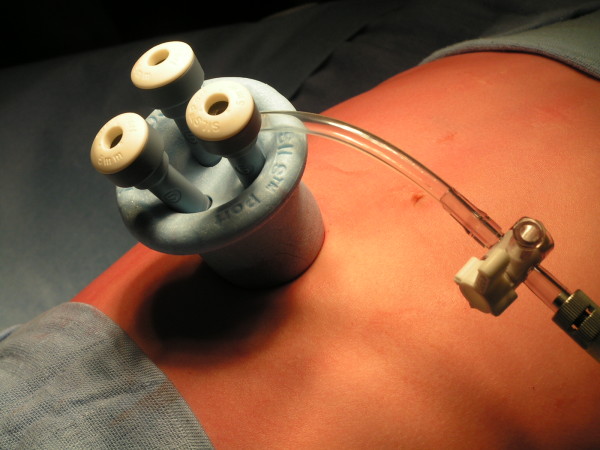
A single-incision laparoscopic surgery port (Covidien) with three inserts was placed into the peritoneal cavity.

**Figure 3:**
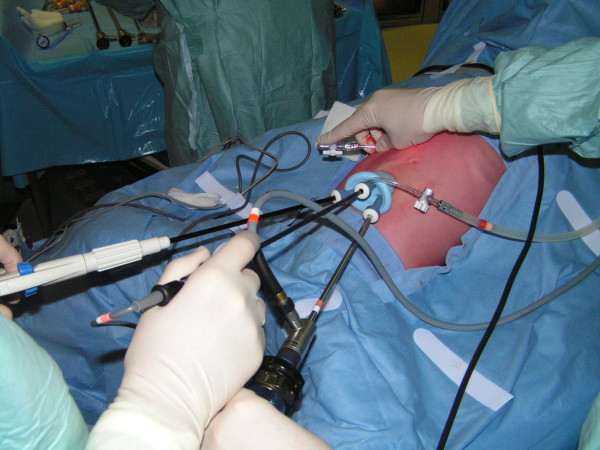
The three ports form an inverted triangular configuration, with the two working ports being farthest away and the laparoscope being at the inferior position.

Under vision, the SILS® port (Covidien, Mansfield, MA, USA), a specially designed single port with three inserts, was placed into the peritoneal cavity (Figure 2). A pneumoperitoneum of 12mmHg was developed. A 5mm laparoscope was used for visualization and two 5mm working instruments were inserted. The three ports formed an inverted triangular configuration, with the two working ports being farthest away and the laparoscope being at the inferior position. The extracorporeal lengths of the ports were kept different, with the laparoscopic port being the longest so as to minimize collisions at the back ends. A standard 30° laparoscope was used for the procedure (Figure 3).

**Figure 4:**
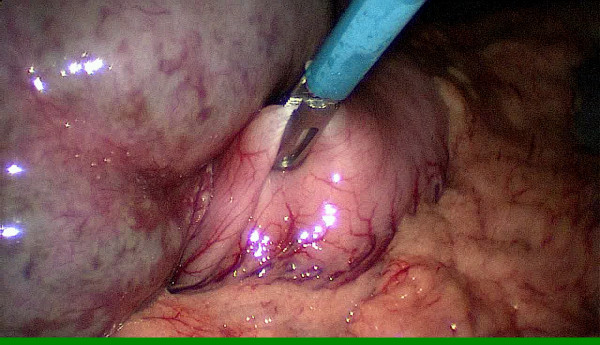
With the help of the instruments the stomach could be identified under the left liver lobe and grasped along the great curvature during the single-incision laparoscopic surgery procedure.

During abdominal exploration with standard straight instruments we could see a very large heterogeneous left liver lobe and an enormous spleen. It was difficult to see the stomach. With the help of the instruments the stomach could be identified under the left liver lobe. We grasped the stomach along the great curvature with an instrument and brought it outside the abdomen by extracting the single port (Figure 4). The gastrostomy passed the skin and abdominal wall in the left upper quadrant and was then grasped and brought outside through the incision of the single port at the umbilicus. Outside the abdomen we made a purse-string suture on the stomach. The gastrostomy was placed in the stomach and the purse-string was closed. We filled the balloon of the gastrostomy with 5cm^3^ of saline solution. Once the gastrostomy was placed in the stomach, the stomach was returned into the abdomen. The fascial defect, and thus also the hernia, was repaired with a Vicryl 2–0 suture (Ethicon, Johnson & Johnson) and the 2cm umbilical incision was closed with endocutaneous sutures.

The total operative time was 25 minutes. There was no blood loss. Our patient’s post-operative course was uneventful, and she required minimal analgesia.

Our patient was able to use the feeding tube on the first post-operative day with good intestinal function.

**Figure 5:**
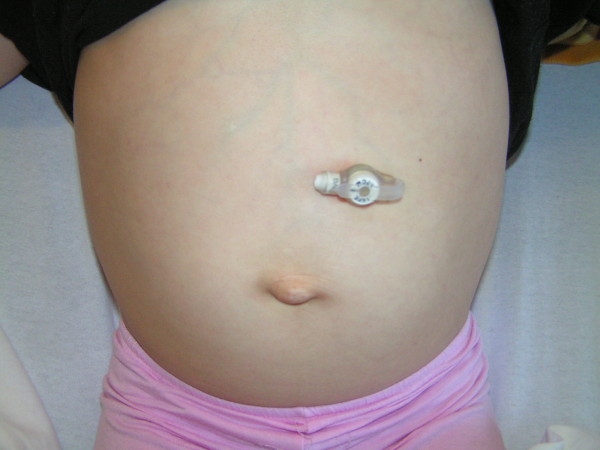
Follow-up at two weeks showed no evidence of complications, and the umbilical wound was healed.

Our patient was discharged on the third post-operative day with no oral analgesics. Our patient and her parents were very pleased with the cosmetic result. Early follow-up at two weeks showed no evidence of complications, and the umbilical wound had healed (Figure 5).

## Discussion

SILS has recently been developed, and has been embraced by many throughout the world as the new paradigm in laparoscopy.

Potential advantages over conventional laparoscopic surgery include better cosmesis, less post-operative wound pain, earlier recovery and fewer port site complications. A SILS procedure at the level of the umbilicus is also less traumatic than the conventional three-trocar laparoscopic approach, because no trocar is inserted into the abdomen through the abdominal muscles, which reduces possible injury to intra-abdominal organs [1]. In SILS, the insertion of the port is performed under direct view.

Most of the reports in the literature on SILS are in adults, such as SILS for appendectomy, cholecystectomy and colectomy and in bariatric surgery. Single-incision laparoscopic appendectomy [2-4] and cholecystectomy [5-7] are SILS procedures that have been described in pediatric patients. Few case reports of splenectomy [8-10] and nephrectomy [11,12] by SILS approach in children have been described to date.

For the placement of a gastrostomy in children, different techniques have been described in the literature. The laparoscopic U-stitch technique, first described by Georgeson in 1993, allows primary button placement and carries the advantages of laparoscopy. It enables direct visualization of the intra-peritoneal anatomy, greatly minimizing the risk of hollow viscous or vascular injury [13]. Kawahara *et al*. reported a single-port technique using a 15mm incision and operating laparoscope [14]. Ponsky *et al*. described a single-site laparoscopic gastrostomy with a 4mm bronchoscopic optical grasp, which is a minimal invasive procedure that provides direct visualization through a single 5mm abdominal port [15].

Via our SILS technique it was possible to explore the whole abdomen, as in the procedures described above, but it was also possible to use extra instruments to push away the liver and spleen to allow optimal visualization of the stomach and perfect intra-peritoneal anatomy.

We recommend the use of conventional laparoscopic instruments to keep the procedure costs equal to conventional laparoscopic surgery.

The cosmetic result of this SILS procedure was excellent, with the scar above the umbilicus. A virtually scar-free surgery is important in pediatric patients, as a scar is a stigma for life. Normally in SILS procedures, we make an incision into the umbilicus in order to make it a scar-free procedure, but in this case we decided to make a supra-umbilical incision to correct the umbilical hernia at the same time.

This early experience suggests that outcomes are comparable to standard laparoscopic surgery, but with improved cosmesis, less pain and earlier recovery; however, larger series should confirm these findings.

Pediatric surgeons need to play an active role in developing the SILS approach for additional procedures, as well as encouraging the industry to produce instruments specific for pediatric needs.

The development of a flexible-tip laparoscope, together with articulated laparoscopic instruments, will make the procedure slightly easier and thus attractive for many pediatric surgeons. With more input from the experience of colleagues around the world, the feasibility and safety of adopting SILS into pediatric practice, as well as its benefits over conventional laparoscopic surgery, could be determined.

## Conclusions

Single-incision surgery is a developing technique in children. In this article we describe our first experience of single-incision gastrostomy in a pediatric patient. We have shown that single-port gastrostomy can be safe and effective in pediatric patients, and may be a replacement for traditional laparotomy for the placement of a gastrostomy.

Furthermore, we hope to extend the use of this operative approach to other commonly performed pediatric procedures.

## Consent

Written informed consent was obtained from the patient’s next of kin for publication of this manuscript and any accompanying images. A copy of the written consent is available for review by the Editor-in-Chief of this journal.

## Competing interests

The authors declare that they have no competing interests.

## Authors’ contributions

KV, NV and KD performed the operation. KV and KD were major contributors in writing the manuscript. AD and GD revised the manuscript. All authors read and approved the final manuscript.
